# Detection of high prevalence of *Plasmodium falciparum* histidine-rich protein 2/3 gene deletions in Assosa zone, Ethiopia: implication for malaria diagnosis

**DOI:** 10.1186/s12936-021-03629-x

**Published:** 2021-02-23

**Authors:** Gezahegn Solomon Alemayehu, Kayla Blackburn, Karen Lopez, Cheikh Cambel Dieng, Eugenia Lo, Daniel Janies, Lemu Golassa

**Affiliations:** 1 Addis Ababa University, Aklilu Lemma Institute of Pathobiology, Addis Ababa, Ethiopia; 2 Department of Bioinformatics and Genomics, University of North Carolina at Charlotte, Charlotte, NC 28223 USA; 3 Department of Biological Sciences, Charlotte, University of North Carolina at Charlotte, Charlotte, NC 28223 USA

**Keywords:** *Plasmodium falciparum*, *Pfhrp2/3* gene deletion, PfHRP2 RDT, Microscopy, PCR, Assosa, Ethiopia

## Abstract

**Background:**

Rapid diagnostic tests (RDTs) targeting histidine rich protein 2(HRP2) are widely used for diagnosis of *Plasmodium falciparum* infections. Besides PfHRP2, the PfHRP3 antigen contributes to the detection of *P. falciparum* infections in PfHRP2 RDTs. However, the performance HRP2-based RDT is affected by *pfhrp2/3* gene deletions resulting in false-negative test results. The objective of this study was to determine the presence and prevalence of *pfhrp2/3* gene deletions including the respective flanking regions among symptomatic patients in Assosa zone, Northwest Ethiopia.

**Methods:**

A health-facility based cross-sectional study was conducted in febrile patients seeking a malaria diagnosis in 2018. Blood samples were collected by finger-prick for microscopic examination of blood smears, malaria RDT, and molecular analysis using dried blood spots (DBS) prepared on Whatman filter paper. A total of 218 *P. falciparum* positive samples confirmed by quantitative PCR were included for molecular assay of *pfhrp2/3* target gene.

**Results:**

Of 218 *P. falciparum* positive samples, exon 2 deletions were observed in 17.9% of *pfhrp*2 gene and in 9.2% of *pfhrp*3 gene. A high proportion of deletions in short segments of *pfhrp2* exon1-2 (50%) was also detected while the deletions of the *pfhrp3* exon1-2 gene were 4.1%. The deletions were extended to the downstream and upstream of the flanking regions in *pfhrp2/3* gene (above 30%). Of eighty-six PfHRP2 RDT negative samples, thirty-six lacked *pfhrp2 exon 2*. Five PfHRP2 RDT negative samples had double deletions in *pfhrp2 exon 2* and *pfhrp3 exon2*. Of these double deletions, only two of the samples with a parasite density above 2000 parasite/µl were positive by the microscopy. Three samples with intact *pfhrp3 exon2* in the *pfhrp2 exon2* deleted parasite isolates were found to be positive by PfHRP2 RDT and microscopy with a parasite density above 10,000/µl.

**Conclusion:**

This study confirms the presence of deletions of *pfhrp2/3* gene including the flanking regions. *Pfhrp2/3* gene deletions results in false-negative results undoubtedly affect the current malaria control and elimination effort in the country. However, further countrywide investigations are required to determine the magnitude of *pfhrp2/3* gene deletions and its consequences on routine malaria diagnosis.

## Background

Despite significant progress made in the last decade towards malaria control and elimination in most malaria-endemic countries in the world, malaria is still a major public health problem [1]. *Plasmodium falciparum* and *Plasmodium vivax* co-exist in Ethiopia [2]. *Plasmodium falciparum* is responsible for most of the malaria-associated deaths in malaria endemic countries. Accurate malaria diagnostic tools and prompt treatment play an important role in reducing morbidity and mortality of malaria [3].

To date, the approach to malaria diagnosis depends on the specific requirements of the malaria program and the dynamics of malaria transmission. Microscopy and rapid diagnostic tests (RDTs) are the most common front-line diagnostic tools in clinical settings. Molecular diagnostic methods are recently used in countries with low-transmission setting and/or approaching malaria elimination phase [4].

Malaria RDTs targeting histidine-rich protein-2 (HRP2) and lactate dehydrogenase (LDH) are commonly used for diagnosis of *P. falciparum* and non-falciparum malaria parasites, respectively. In Ethiopia, where *P. falciparum* predominates, PfHRP2 RDT is widely used [5]. However, a number of factors would affect the performance of PfHRP2 RDT. Among others, the lack of HRP2 antigen production in *P. falciparum* parasites due a deletion of parts or all of the *pfhrp2* gene [6], affects the sensitivity and specificity of HRP2 RDTs. Besides, PfHRP2, the PfHRP3 antigen produced by *P. falciparum* has been shown to share high homology with PfHRP2 [7]. This PfHRP3 antigen may contribute to the detection of *P. falciparum* infections in PfHRP2 RDTs, but the performance of these RDTs could also be affected if there is a deletion of the *pfhrp3* gene in *P. falciparum* [8].

Interestingly, partial or entire deletion of *pfhrp2* and *pfhrp3* genes in *P. falciparum* populations could result in false negative RDT-based diagnosis of malaria [6], which would delay timely prescription of anti-malarial drugs and allow the rapid spread of these genotypes from untreated patients into the communities. Therefore, *P. falciparum* populations containing these variants of the *pfhrp2* and *pfhrp3* genes would be selected and would remain a major threat to existing malaria case management and control efforts [9].

In recent years, deletion of *pfhrp2* and/or *pfhrp3* genes have been reported in different geographical regions including the Peruvian Amazon Basin [10], Brazil [11], India [12], and Senegal [13]. The effect of *pfhrp2* and *pfhrp3* gene deletion on the performance of PfHRP2RDTs have also been evaluated in a few East African neighbouring countries of Ethiopa, such as Eritrea [14], Sudan [15], and Kenya [16]. Therefore, it is very important to explore the presence and prevalence of *pfhrp*2/3 gene deletions in the Ethiopian *P. falciparum* populations. Moreover, there are no reports of *pfhrp2/3* genes deletions in Assosa zone in Northwest Ethiopia. Thus, objective of this study was to assess the presence and prevalence of *pfhrp2/3* genes deletion as well as the respective flanking regions among *P. falciparum* isolates in this area.

## Methods

### Study area

**Fig. 1:**
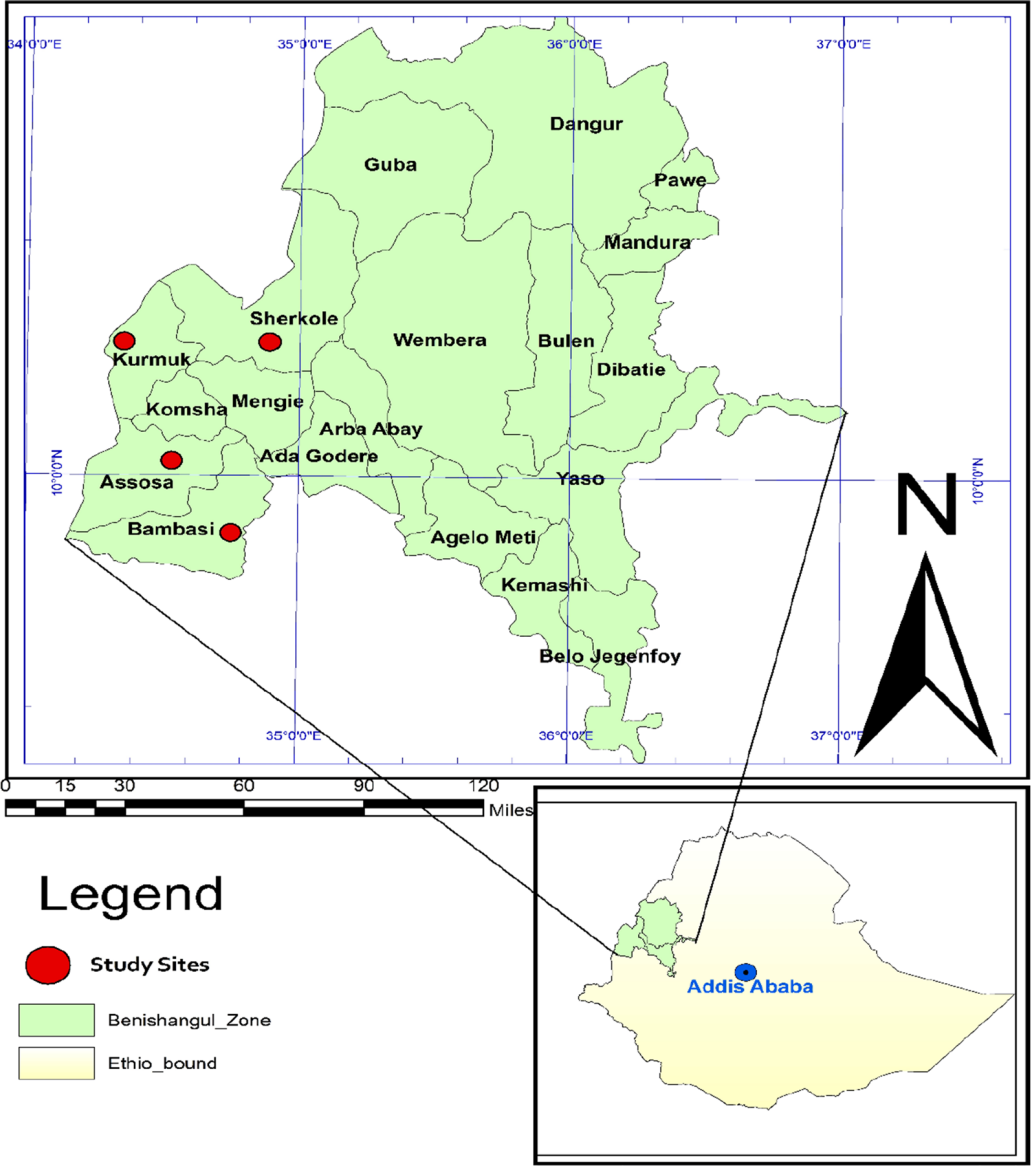
The map showing the study area in Assosa zone. The map generated using ArcGIS version 10.0 software

A cross-sectional study was conducted in clinical setting during low and high transmission seasons at four selected health facilities: Assosa, Bambasi, Kurmuk and Sherkole Health Centres in Assosa Zone, Benishangul-Gumuz Regional State, Northwest Ethiopia from November to December 2018. The study area was selected using a simple random sampling technique among eight districts in the Assosa zone. Most of the district has a high intensity of transmission according to the annual parasite incidence report [2]. Majority of the population of the districts are permanent residents and depends mainly on agricultural and mining work. The study area is located on the northwestern border of Ethiopia where there is a local cross-border market between the people of Sudan and Ethiopia. In line with this, there is import and export of the malaria parasite between the two countries. Recently, there is a confirmed report of the *pfhrp2/3* gene deletion in Sudan [15]. Thus, this study was design to assess the presence and magnitude of the *pfhrp2/3* gene deletion on the northwestern border of Ethiopia. The geographic locations of the study sites was taken by handheld GPS (Garmin GPS 73, United State) and the map was generated using ArcGIS version 10.0 software (Fig. 1).

### Blood sample collection

Demographic data were collected at the time of enrollment from clinically suspected malaria patients. Simultaneously, in a single finger-prick, capillary blood sample was collected from each study participant for thin and thick blood smear preparation for microscopy, and Malaria RDT. Dried blood spots (DBSs) were spotted on Whatman™ FTA™ filter paper (GE Healthcare, Piscataway, NJ, USA) for molecular assay.

### Detection of *P. falciparum* by microscopy and malaria RDT

Thin and thick blood smears were prepared and stained with Giemsa stain working solution diluted in 10% buffer for microcopy detection of *Plasmodium* parasites. Briefly, Giemsa working stain solution prepared by a stock volume of Giemsa with nine volumes of buffered water with pH 7.2. Thin blood smear fixed with methanol solution and staining with Giemsa working stain for 10 min on the staining rack. Thin smears were used to identify *Plasmodium* parasites species while the thick smears were examined to detect and measure the density of the parasite against 200 white blood cells (WBC), assuming mean WBC count was 8000/μL, as per WHO recommendations [17]. The Care Start™ malaria RDT (Pf/PV (HRP2/PLDH, RMVM-02591) was performed and interpreted based on the manufacturer’s instructions in the package insert.

### DNA extraction and quantitative PCR

Genomic DNA was extracted from blood DBS using Chelex-Saponin method as described previously [18]. *Plasmodium falciparum* identification were confirmed by SYBR green based quantitative PCR (qPCR) assay by targeting the 18S rRNA gene using species-specific primers as described elsewhere [19]. Absolute quantification approach was used to quantify unknown samples by qPCR. Serially diluted known quantity of parasite DNA amplified to generate a standard curve. Then, quantifications were performed by comparing Ct-value for unknown samples against this a standard curve.

### Amplification of *pfhrp2* and *pfhrp3* genes

Of the 499 malaria suspected individuals selected for molecular analysis, a total of 218 qPCR confirmed *P. falciparum* positive samples with Ct-value < 37 were included for *pfhrp2/3* molecular analysis to minimize false negative finding of *pfhrp2/3* associated with low density infection in this study (Additional file 1). This study protocol included both the positive and negative samples by microscopy and PfHRP2 RDT to reduce underestimation of *pfhrp2/3* gene deletion due to submicroscopic samples.

After confirmation of *P. falciparum* positive samples by qPCR, *pfhrp2* and *pfhrp3* gene analysis was performed by amplification of two amino acids coding segments (*exon2* and *exon1-2*). The short segment, *exon1-2*, spans the end of *exon1* to the start of *exon2*. The longest segment, *exon 2*, consists of the entire *exon2* which is the main amino acid coding region of *pfhrp2* and *pfhrp3* genes. PCR amplifications were performed on upstream *(PF3D7_0831900 (MAL7P1_230)* and downstream *(PF3D7_0831700 (MAL7P1_228)* flanking region of *pfhrp2* gene. Likewise, PCR amplifications were also performed on upstream *PF3D7_1372100 (MAL13P1_475)* and downstream *(PF3D7_1372400 (MAL13P1_485)* flanking regions of *pfhrp3 gene.* The study flow chart for molecular analysis of *pfhrp*2 and *pfhrp*3 is indicated in Additional file 1.

PCR amplification of *pfhrp2, pfhrp3* genes and their respective flanking regions were carried out in the total reaction mixture of 25 μl using 2X Go Taq hot start green master mix (Integrated DNA Technologies, Inc, Coraville, USA). 0.4 μM each of forward and reverse primer, 3 μl of template DNA for each *pfhrp2 exon2, pfhrp3 exon2* and *pfhrp3 exon1-2*, 2 μl of template DNA for each *pfhrp2 exon1-2*, *MAL7P1_230* and *MAL13P1_475*, 1 μl of template DNA for each *MAL13P1_485* and *MAL7P1_228* were used based on PCR conditions and primer sequences as described previously [9, 20, 21]. As a positive control, 3D7 (MRA-102G) was used for PCR amplification of *pfhrp2, pfhrp3* and their respective flanking genes. Likewise, DNA of laboratory strains Dd2 (MRA-150G) and HB3 (MRA155G) were used as negative control for the PCR amplifications of *pfhrp2* and *pfhrp3* including their flanking genes, respectively [10]. All *pfhrp2/3* PCR negative samples were repeated to confirm the absence of the target gene (*pfhrp2/3* exon 2 and exon 1–2) including flanking regions.

The amplified fragments of *pfhrp2, pfhrp3* and their flanking genes were separated by electrophoresis on a 2% agarose gel by staining with SYBR safe in a Tris Borate EDTA (TBE) buffer. The DNA bands were visualized in a UV transilluminator and the expected amplicons size compared to 1 kb DNA ladder. Primer sequences, PCR conditions and expected amplicon size of *pfhrp2/3 genes* are indicated in Additional file 2.

### Data analysis

The proportion of *pfhrp2 /3* gene deletions were analysed using Statistical Package for Social Sciences (SPSS) version 20. *P* value < 0.05 were considered as significant.

## Results

### qPCR, microscopy and PfHRP2 RDTs

A total of 218 *P. falciparum* positive samples by qPCR were included based on inclusion criteria for molecular analysis of *pfhrp2, pfhrp3 gene* and their flanking gene. Of the study participants positive for *P. falciparum* by qPCR, 56% (122/218) and 62.8% (137/218) of the positive cases were female and in the youngest age group (5–24 years), respectively. PfHRP2 RDT and microscopy detected approximately 60% of infections that were detectable by qPCR. There is statistical difference in positivity rate of PfHRP2 RDTs with respect to gender (*P* = 0.028) and age group (*P* = 0.05) (Table 1).

**Table 1:** Demographic data of *P. falciparum* positive study participants by laboratory test

Variables	Number of positive samples by Laboratory test		
	qPCR	Microscopy	PfHRP2 RDT
	No (%)	No (%)	No (%)
Sex			
Male	96 (44.0)	62 (64.6)	66 (68.8)
Female	122 (56%)	69 (56.6)	66 (54.1)
*P*-value*٭		*P* = 0.230	*P* = 0.028
Age groups			
5–14	56 (25.7)	42 (75.0)	43 (76.8)
15–24	81 (37.2)	50 (61.7)	47 (58.0)
25–34	47 (21.6)	23 (48.9)	23 (48.9)
35–44	19 (8.7)	9 (47.4)	11 (57.9)
45–57	15 (6.9)	7 (46.7)	8 (53.3)
*P *value		*P* = 0.038	*P* = 0.051
Study site			
Sherkole	94 (43.1)	63 (67.0)	63 (67.0)
Bambasi	66 (30.3)	43 (65.2)	46 (69.7)
Kurmuk	42 (19.3)	20 (47.6)	19 (45.2)
Assosa	16 (2.8)	5 (31.3)	4 (25.0)
*P *value		*P* = 0.013	*P* = 0.001

*No* Number, *% *percent, *Pf Pos* *P. falciparum* Positive

*٭Statistically significant at p-value less than 0.05

### Deletions in* pfhrp2* and *pfhrp3 genes*

Among the 218 *P. falciparum* positive isolates, exon 2 deletions were observed in 17.9% (39/218) of *pfhrp*2 gene and in 9.2% (20/218) of *pfhrp*3 gene. Likewise, deletions of the *pfhrp2 exon1-2* gene exhibited in 50% (109/218) of the isolates while deletions of the *pfhrp3 exon1-2* gene were 4.1% (9/218). The deletions extended to upstream and downstream of the flanking genes of both *pfhrp2 (MAL7P1_230* and *MAL7P1_228)* and *pfhrp3 (MAL13P1.475* and *MAL13P1.485)* accounting for 40% of the deletions. The deletion pattern of *Pfhrp2/3* and its flanking region was varied in different isolates of *P. falciparum.* Of the 218 *P. falciparum* isolates, 37.2% of the isolates did not have deletions in the *pfhrp2/3* gene, including its flanking regions (Additional file 3).

The pattern of deletions in *pfhrp2/3* genes and their flanking regions varies according to the study sites. The high proportion of deletions of the *pfhrp2 exon2* gene were observed in 37.5% (6/16) of the isolates collected at the Assosa health centre (Table 2).

**Table 2:** PCR negative results of *pfhrp2* and *pfhrp3* genes including flanking regions among *P. falciparum* positive isolates

Study site	Pf pos samples by qPCR	*Pfhrp2-exon2*	*Pfhrp2-exon1-2*		*MAL7P1_228*	*MAL7P1_230*	*Pfhrp3-exon2*	*Pfhrp3-exon1-2*		*MAL13P1_475*	*MAL13P1_485*
		Neg	Neg		Neg	Neg	Neg	Neg		Neg	Neg
	No	No (%)	No (%)		No (%)	No (%)	No (%)	No (%)		No (%)	No (%)
Sherkole	94	14 (14.9)	48 (51.1)		53 (56.4)	42(44.7)	8(8.5)	5 (5.3)		51 (54.3)	51 (54.3)
Bambasi	66	9 (13.6)	23 (34.8)		28 (42.4)	20 (30.3)	6 (9.1)	2 (3.0)		25 (37.9)	27 (40.9)
Kurmuk	42	10 (23.8)	26 (61.9)		28 (66.7)	22 (52.4)	5 (11.9)	2 (4.8)		26 (61.9)	28 (66.7)
Assosa	16	6 (37.5)	12 (75)		11 (68.7)	9 (56.3)	1 (6.3)	0 (0)		11 (68.8)	11 (68.8)
Total	218	39 (17.9)	109 (50)		120 (55.1)	93 (42.7)	20 (9.2)	9 (4.1)		113 (51.8)	117 (53.7)

Pf Pos=*P. falciparum, *Positive, *Neg* Negative

### *Pfhrp2/3* genes deletions by transmission seasons

**Fig. 2:**
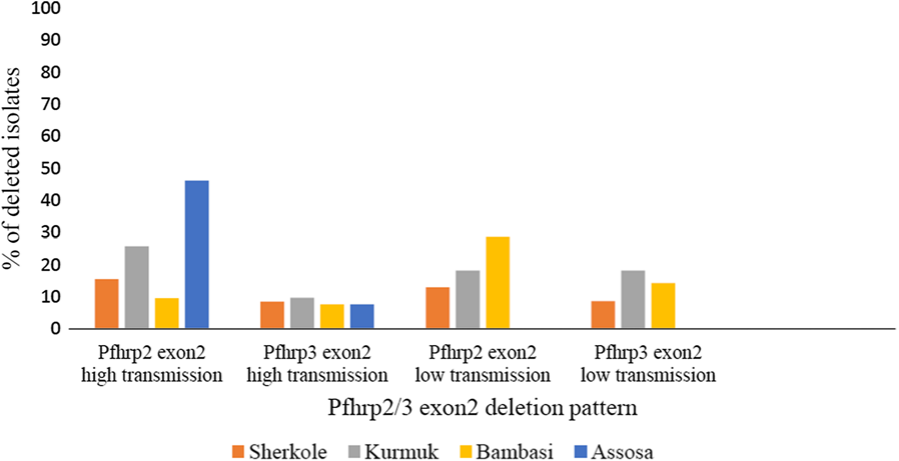
*Pfhrp2/3 exon2* deletion pattern during high and low transmission seasons by the study site

A comparable *pfhrp2/3* genes deletions were observed in both high and low transmission seasons. The frequency of *pfhrp*2 exon 2 deletion was 19.9% (30/167) in high transmission season and 17.7% (9/51) in the low transmission season. The frequency of *pfhrp3 exon 2* deletion was 8.4% (14/167) and 11.8% (6/51) in high and low transmission seasons, respectively. The extent of deletions of *pfhrp2/3* exon2 varied by the study site. High proportion deletion of *pfhrp2* exon2 (46.2%) was observed in the Assosa district during the high transmission season followed by 28.6% of *pfhrp2* exon2 deletion in Bambasi district during low transmission season (Fig. 2).

### PfHRP2 RDT and microscopy compared to PCR results of *pfhrp2 and pfhrp3*

**Fig. 3:**
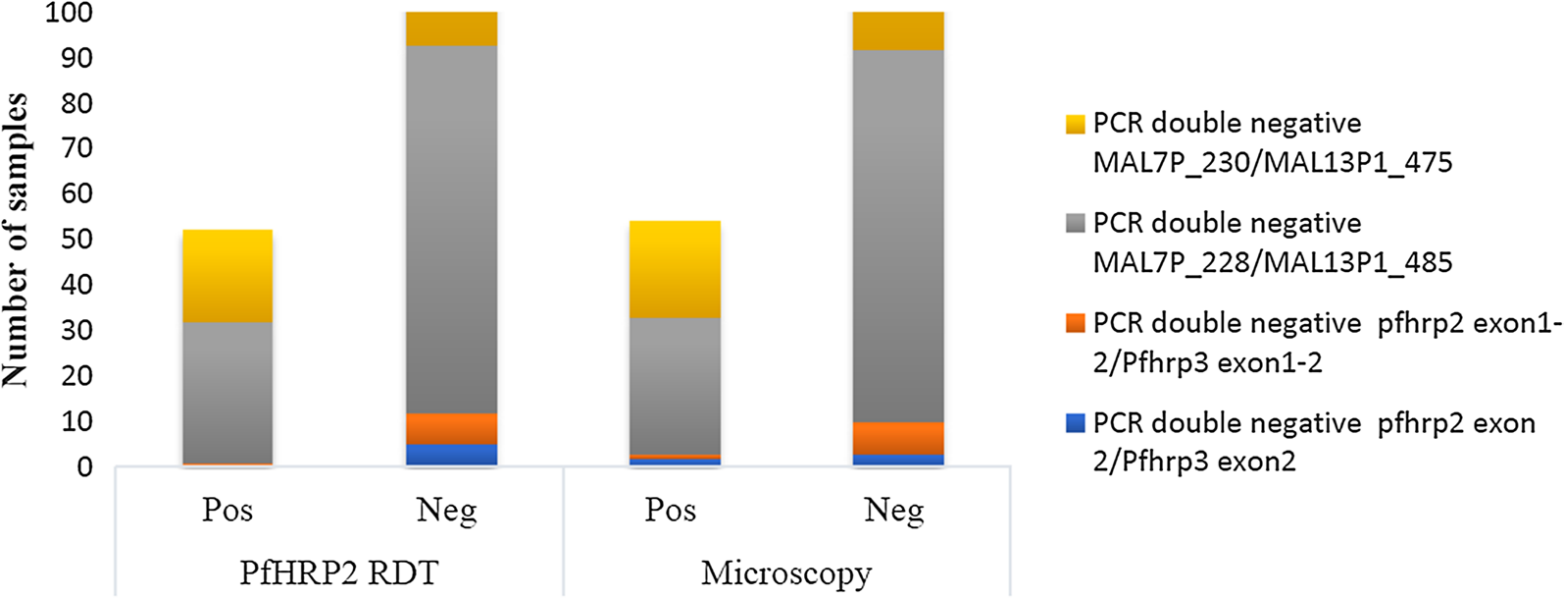
PfHRP2 RDT and Microscopy results associated with PCR double negative results of *Pfhrp2/3* genes including flanking region among *P. falciparum* positive isolate. Note: Upstream *(MAL7P1_230)* and downstream *(MAL7P1_228)* flanking regions of *Pfhrp2*. Upstream (*MAL13P1_475*) and downstream *(MAL13P1_485)* flanking regions of *pfhrp3*

Of eighty-six PfHRP2 RDT negative samples, 41.9% (36/86) samples lacked *pfhrp2 exon 2* as compared to 10.5% (9/86) deletions observed in *pfhrp3 exon* 2 (Table 3). Of thirty-six *pfhrp2 exon 2* PCR negative samples, thirty-three samples were submicroscopic unlike the three samples with the parasite densities above 1000 /µl (Additional file 4). A total of five PCR double negative samples for *pfhrp2 exon 2* and *pfhrp3 exon*2 gene were also PfHRP2 RDT negative (Fig. 3)*.* Among those five PCR double negative results of *pfhrp*2 *exon 2* and *pfhrp3 exon*2 gene*,* two PfHRP2 RDT negative samples with parasite density above 2000 parasite/µl were microscopy positive while three of them were submicroscopic (Table 4). On the other hand, three *pfhrp*3 exon2 PCR positive isolates lacked the *pfhrp*2 exon2 gene. These three samples had a parasite density greater than 10,000 / µl and were positive both by PfHRP2 RDT and microscopy in the absence of the *pfhrp*2 gene, suggesting the contribution of intact *pfhrp*3 *exon*2 to reduce false negative PfHRP2 RDT despite a deletion in the *pfhrp*2 gene (Additional file 5).

**Table 3:** PfHRP2 RDT and Microscopy results against PCR results of *pfhrp2 *and *pfhrp3*

PCR results	PfHRP2 RDTs		Microscopy	
	No positive	No negative	No positive	No negative
*Pfhrp2 exon 2*				
Positive	129	50	125	54
Negative	3	36	6	33
*Pfhrp3 exon 2*				
Positive	121	77	117	81
Negative	11	9	14	6
*Pfhrp2 exon1-2*				
Positive	103	6	104	5
Negative	29	80	27	82
*Pfhrp3 exon1-2*				
Positive	130	79	129	80
Negative	2	7	2	7

**Table 4:** Parasite densities and PfHRP2 RDT results among PCR double deletions of *pfhrp2 exon2* and *pfhrp3 exon2* genes

Sample ID	Parasite density/µl as determined by microscopy	Parasite density/µl as determined by qPCR	PfHRP2 RDT results	*Pfhrp2 gene* including the flanking regions				*Phrp3 gene* including the flanking region			
				*MAL7P1_230*	*Pfhrp2 Exon1-2*	*Pfhrp2 Exon2*	*MAL7P1_228*	*MAL13P1_475*	*Pfhrp3 Exon1-2*	*Pfhrp3 exon2*	*MAL13P1_485*
HShr154	4800	1406.49	−	−	−	−	−	−	−	−	−
Hkum68	__	23,827,545	−	−	−	−	−	−	+	−	−
LShr106	__	134.61	−	−	−	−	−	−	−	−	−
LBa16	__	999.1	−	−	−	−	−	−	+	−	−
Lkum59	10,000	773.06	−	−	−	−	−	−	+	−	−

+  = Positive,,− = Negative,. Upstream *(PF3D7_0831900(MAL7P1_230)* and downstream *(PF3D7_0831700(MAL7P1_228)* flanking regions of *Pfhrp2*. Upstream *(PF3D7_1372100(MAL13P1_475))* and*)* downstream *(PF3D7_1372400(MAL13P1_485))* flanking regions of *pfhrp3*

Regarding exon1-2 of *pfhrp*2/3, a high proportion of negative samples of PfHRP2 RDT lacked exon1-2 of *pfhrp*2 (93%, 80/86) compared to exon 1–2 of *pfhrp*3 (8.1%, 7/86). Out of eighty *pfhrp*2 exon1-2 PCR negative samples, only five samples were tested positive for microscopy (Table 3). Furthermore, among eight PCR double negative results for *pfhrp2* exon1-2 / *pfhrp3* exon1-2, seven samples were negative for both PfHRP2 RDT and microscopy (Fig. 3).

### PfHRP2 RDT results against PCR results of *pfhrp*2/3 flanking regions

Out of eighty-six PfHRP2 RDT negative samples, more than 80% of the samples lacked *MAL7P1_230, MAL13P1_475, MAL7P1_228* and *MAL13P1_485* in both the upstream and downstream *pfhrp2/3* flanking region (Additional file 6). Out of eight-six double deletions of MAL7P1_230 and MAL13P1.475, sixty-six negative samples were found by PfHRP2 RDT and sixty-five negative samples by microscopy. Of one hundred twelve double deletions of MAL7P1_228 and MAL13P1.485, a total of eighty-one samples by PfHRP2 RDT and eighty-two samples by microscopy were negative (Fig. 3).

## Discussion

This study confirmed the presence of a considerable number of *pfhrp*2/3 gene deletions in *P. falciparum* clinical isolates in the Assosa zone, Northwest Ethiopia. Partial deletions of *pfhrp*2*, pfhrp3,* and the flanking genes were observed in the majority of the parasite populations while complete deletion of *pfhrp*2/3 gene occurred only in two of *P. falciparum* clinical isolates. On the other hand, 37.2% (81/218) of the clinical isolates did not have deletion in exon 2 and exon 1–2 of *pfhrp*2/3 region including the flanking regions.

Of 218 positive samples for *P. falciparum*, microscopy and RDT were able to detect almost 60% of the infections detected by qPCR. If these large number of false negatives individual remain undetected and untreated, they could likely serve as malaria reservoirs and accelerate the onward transmission of malaria in the community[22]. High proportion of PfHRP2 RDT false-negative results in this study is suggested to be due to the deletion of *pfhrp2/3* gene in the *P. falciparum* populations.

The prevalence of *pfhrp2* exon2 deletion in this study (17.9%; 39/218) varied with the findings reported by previous studies such as comparable results in Kenya [16], lower results in Ethiopia [23], Mozambique [24], China [25], and higher than the current study in Ethiopia [26], Eritera [14] and Sudan [15]. Likewise, the *pfhrp3 exon2* deletion (9.2%; 20/218) in this study was higher than study in Kenya [16], but lower than deletions reported from Ethiopia [26], Eritera [14], Ghana [27], and Honduras [21]. Regarding transmission season, a comparable number of *pfhrp*2/3 genes deletions were observed in both high and low transmission seasons in this study area. This finding contrast with other studies in Rwanda [28] and Zanzibar [29]. The possible explanation for these differences might be due to variation in transmission intensity [28, 30], host immunogenic response [31, 32], drug used [33], geographical locations [8, 34], sample size, and laboratory methods [35–37] used to analyse *pfhrp*2/3 genes deletions.

Out of a total of 39.5% (86/218) PfHRP2 RDT negative samples in this study, a high proportion of PCR confirmed deletions were observed in *pfhrp2* /3 *exon2* and *exon1-2*, and upstream and downstream flanking regions of *pfhrp*2/3 compared to WHO *pfhrp*2/3 deletion threshold level [38]. Approximately forty-two percent of (36/86) PfHRP2 RDT negative samples lacked *exon 2* of *pfhrp2*, the main amino acid coding region of the *pfhrp2* gene. Of these PCR negative *Pfhrp2* exon 2 samples, only three of the samples with a parasite density above 1000 / µl were microscopic positive while the rest were submicroscopic. Moreover, high prevalence of deletion was detected in short segmented of *pfhrp2 exon1-2*(50%) and *pfhrp*3 *exon1-*2(4.1%). High proportion of deletions (> 30%) was also extended to the downstream and upstream of the flanking regions in *pfhrp2/3* gene. The complete and partial *pfhrp*2/3 genes deletions observed in this study would undoubtedly impact the diagnostic performance of PfHRP2 RDTs.

According to the WHO protocol, a deletion of the *pfhrp2/3* gene in a certain region will be suspected if there is an increase in discordant reports between routine diagnostic tools, such as microscopy positive and PfHRP2 RDT negative [9]. The protocol for this study reiterates that submicroscopic infections should not be overlooked when it comes to *pfhrp2/3 gene* deletions. Therefore, positive and negative microscopic results were included in this study to reduce underestimation of the true prevalence of *pfhrp2/3* gene deletion due to submicroscopic infection. As a result, thirty-three samples with the *pfhrp2* deletion gene were found to be submicroscopic in this study and included only those samples with a Ct value below 37 using qPCR (and hence had > 9 parasite density/µl) to minimize the risk of finding false deletion results associated with a single copy of the target gene (*Pfhrp2*) and low-density infection. *P. falciparum* positive clinical isolates with *pfhrp2/3 gene* deletion may selectively increase if there is no continuous monitoring associated with submicroscopic infections and PfHRP2 RDT negative results. Those parasites with submicroscopic parasite densities with *pfhrp2/3* gene deletions could be potentially infectious to mosquitoes and contribute to ongoing malaria transmission which could in turn challenge malaria control and prevention.

In this study, 58.1% (50/86) false negative PfHRP2 RDT results were found to be *pfhrp2* exon2 positive by PCR. The likely cause of these false negative PfHRP2 RDT results could be due to low levels of parasitaemia that could be below the detection threshold of PfHRP2 RDT in those submicroscopic infections and absence of PfHRP2 antigen due to host immune response in the high transmission areas [39].

All PCR double negative samples in *pfhrp*2/3 genes and their flanking regions were PfHRP2 RDT negatives in this study. The number of PCR double negative samples of *pfhrp2 exon 2* and *pfhrp*3 *exon* 2 were low compared to previous studies in Peru [10] and Ghana [27]. In the reported study, discordant results between PfHRP2 RDT and microscopy were observed in five PCR double negative samples of *pfhrp*2 *exon* 2 and *pfhrp*3 *exon 2*. Two PfHRP2 RDT negative samples were microscopic-positive with a parasite density above 2000 parasite/µl while the remaining three samples were submicroscopic. The presence of double deletions of *pfhrp*2/3 genes in this study would indicate the potential challenge of PfHRP2 RDT based malaria diagnosis in the study area.

The contribution of intact *pfhrp*3 gene to PfHRP2 RDT positive results is noted in the study under report. Even though only three *pfhrp3 exon2* PCR positive samples were lacking *pfhrp2 exon2,* these three samples were confirmed positive by PCR and microscopy with parasite density above 10,000/µl in the absence of *pfhrp2* gene suggesting the contribution of *pfhrp3 exon2.* This finding indicated that the structural homology between epitopes of PfHRP2 and PfHRP3 antigens allows cross-reaction of the monoclonal antibodies of PfHRP2 with PfHRP3 [40], which is in agreement with the previous studies [16, 27]. Indeed, PfHRP2 RDT positive test result with intact *pfhrp3* exon2 might underestimate the true prevalence of *pfhrp2* gene deletion in this study.

However, it is yet unclear what causes partial or complete deletion of *pfhrp2/3* gene in *P. falciparum* isolates in Assosa zone in Ethiopia. Previous studies have indicated that deletion of *pfhrp2* in Dd*2* and *pfhrp3* genes in HB3 laboratory strains was resulted from deletion at distinct and unlinked positions on chromosome 8 and 13, respectively [10, 41, 42]. Thus, selection may act differently on the two gene regions leading to different frequency of deletions. A possible explanation for the presence of a considerable number of deletions of *pfhrp2/3* gene in this region may be the selective pressure of *P. falciparum* isolates with *pfhrp2* deletion. This study is located in the Assosa zone, on the border with Sudan, which has a high transmission of malaria. Consequently, intense and routine screening of malaria with a large number of RDTs may pose selective pressure on *P. falciparum* and results in a considerable number of parasites carrying *pfhrp2/3* gene deletion in this region. Furthermore, in the high transmission setting, polyclonal infection is common. *pfhrp2/3* deleted and non-deleted strains may co-exist within an infected individual and result in positive PfHRP2 RDT [33]. Future study merits expanded samples covering broader geographic regions of Ethiopia and in-depth investigations for the detection of *pfhrp2/3* deletion in polyclonal infections of *P. falciparum* using a multiplex qPCR and its effect on malaria diagnosis. Further investigations of the PfHRP2 antibodies in the plasma of *P. falciparum* patients infected with *pfhrp2/3* deleted and non-deleted strains will clarify the impact of gene variation on the host immunogenic responses.

## Limitations

This study has certain limitations. Measurement of PfHRP2 antigen in the plasma was not performed. The molecular methods of this study could not show *pfhrp2/3* gene deletion in polyclonal infections of *P. falciparum.* This study was conducted in a limited geographic area.

## Conclusion

This study confirms the presence of more than 5% of *pfhrp2/3* gene deletions in *P. falciparum* isolates in the study area according to the WHO protocol [9]. The high proportion of PfHRP2 RDT false-negative results due to the deletion of *pfhrp2/3* gene could affect malaria control and elimination efforts in the country. Hence, further country-wide assessment on the magnitude of *pfhrp*2/3 deletions is important before considering alternative malaria diagnostic strategies.

## Supplementary Information

**Additional file 1**: Study flow chart for molecular analysis pfhrp2 and pfhrp3 gene.

**Additional file 2**: Primer sequences, PCR conditions and expected amplicon sizes of Pfhrp2 and Pfhrp3.

**Additional file 3**: Deletion pattern of Pfhrp2/3 and their respective flanking regions among 218 samples.

**Additional file 4**: The results of PCR Pfhrp2 exon2, PfHRP2 RDTs, Microscopy with Parasite density /µl, and qPCR with parasite density/μl and Ct values among P.falciparum positive isolates.

**Additional file 5**: Pfhrp2 exon2 negatives, pfhrp3 exon2 , PfHRP2 RDT and microscopy positive samples.

**Additional file 6**: PfHRP2 RDT and microscopy-based results against PCR-based results of pfhrp2 and pfhrp3 their flanking regions.

## Data Availability

The datasets used and/or analysed during in this study are included in this published article and available from the corresponding author on reasonable request.

## Abbreviations

HRP2: Histidine-Rich Protein 2
PfHRP 2: Plasmodium *falciparum* Histidine-Rich Protein 2
RDT: Rapid diagnostic test
SPSS: Statistical Package for Social Sciences
WHO: World Health Organization
DBS: Dried blood spots
PCR: Polymerase chain reaction
qPCR: Quantitative polymerase chain reaction
FMOH: Federal Ministry of Health of Ethiopia
