# Supplementary material for: Detection of high prevalence of Plasmodium falciparum histidine-rich protein 2/3 gene deletions in Assosa zone, Ethiopia: implication for malaria diagnosis
Source: Malar J. 2021 Feb 23;20:109. doi: 10.1186/s12936-021-03629-x (PMC8095343; doi:10.1186/s12936-021-03629-x)
Supplement: Supplementary file 1 — Additional file 1: Study flow chart for molecular analysis pfhrp2 and pfhrp3 gene. [file 12936_2021_3629_MOESM1_ESM.docx]

Total no of samples selected for molecular analysis (n =499)

*P. falciparum(Pf)* positive samples confirmed by qPCR(≥5parasite/µl)

PCR Amplification of *pfhrp2 and pfhrp3* gene (n=218)

PCR Amplification of *Pfhrp2 gene* (n=218)

PCR Amplification of *Pfhrp3 gene* (n=218)

*MAL7P1_230* PCR(n=218)

*Pfhrp2 exon1-2* PCR (n=218)

*Pfhrp2 exon 2* PCR (n=218)

*MAL7P1_218* PCR(n=218)

*MAL13P1_475* PCR (n=218)

*Pfhrp3 exon1-2* PCR (n=218)

*Pfhrp3 exon 2* PCR (n=218)

*MAL13P1_485*PCR (n=218)

Total of 218 Pf Positive samples selected with Ct–value <37

Additional file 1: Study flow chart for molecular analysis *pfhrp2 and pfhrp3 gene*.
