# Supplementary material for: Detection of high prevalence of Plasmodium falciparum histidine-rich protein 2/3 gene deletions in Assosa zone, Ethiopia: implication for malaria diagnosis
Source: Malar J. 2021 Feb 23;20:109. doi: 10.1186/s12936-021-03629-x (PMC8095343; doi:10.1186/s12936-021-03629-x)
Supplement: Supplementary file 2 — Additional file 2: Primer sequences, PCR conditions and expected amplicon sizes of Pfhrp2 and Pfhrp3. [file 12936_2021_3629_MOESM2_ESM.docx]

| **Additional file 2:** Primer sequences, PCR conditions and expected amplicon sizes of *Pfhrp2* and *Pfhrp3* | | | | | |
| --- | --- | --- | --- | --- | --- |
| **Gene** | **Primer** | **Primer sequences (5′→3′)** | **PCR Condition** | **Amplicon size** | **Reference** |
| PF3D7_0831800 | Pfhrp2_ex2_F | ATT CCG CAT TTA ATA ATAACT TGT GTAGC | 95 °C × 15 min;40 cycles of 94 °C × 1 min, | 600-960bp | [21] |
| (Pfhrp2 exon2) | Pfhrp2_ex2_R | ATG GCG TAG GCA ATGTGT GG | 59 °C × 1 min,72 °C × 1 min;72 °C × 10 min |  |  |
| PF3D7_0831800 | Pfhrp2_ex1-2_F | GGT TTC CTT CTC AAA AAA TAA AG | 94ºC x 5 min; 30 cycles of 94ºC x 30 sec, | 303bp | [21] |
| ( Pfhrp2 exon1-2) | Pfhrp2_ex1-2_R | CTA CAC AAG TTA TTATTA AAT GCG GAA | 55ºC x 50 sec, 72ºC x 1min; 72ºC x 5min |  |  |
| PF3D7_0831900 | Pfhrp2_flak230_F | TAT GAA CGC AAT TTA AGT GAG GCA G | 94ºC x 5 min, 40 cycles of 94ºC x 30 sec, | 346bp | [9] |
| (MAL7P1_230) | Pfhrp2_flak230_R | TAT CCA ATC CTT CCT TTG CAA CAC C | 64ºC x 50 sec, 72ºC x 1min; 72ºC x 5min |  |  |
| PF3D7_0831700 | Pfhrp2_flak228_F | AGA CAA GCT ACC AAA GAT GCA GGT G | 94ºC x 5 min, 30 cycles of 94ºC x 30 sec, | 227bp | [9] |
| (MAL7P1_228) | Pfhrp2_flak228_R | TAA ATG TGT ATC TCC TGA GGT AGC | 60ºC x 50 sec, 72ºC x 1min; 72ºC x 5min |  |  |
| PF3D7_1372200 | Pfhrp3_ex2_F | AAT GCA AAA GGA CTT AAT TC | 95 °C × 15 min; 40 cycles of 94 °C × 1 min, | 600-650bp | [21] |
| (Pfhrp3 exon 2) | Pfhrp3_ex2_R | TGG TGT AAG TGA TGC GTA GT | 55 °C × 1 min, 60 °C × 1 min; 60 °C × 10 min |  |  |
| PF3D7_1372200 | Pfhrp3_ex1-2_F | TAT CCG CTG CCG TTT TTG CTT CC | 95 °C × 15 min; 40 cycles of 94 °C × 1 min | 301bp | [21] |
| (Pfhrp3 exon 1-2) | Pfhrp3_ex1-2_R | TGC ATG ATG GGC ATC ACC TG | 60 °C × 1 min, 60 °C × 1 min; 60 °C × 10 min |  |  |
| PF3D7_1372100 | Pfhrp3_flak475_F | TTC ATG AGT AGA TGT CCT AGG AG | 94ºC x 5 min, 30 cycles of 94ºC x 30 sec, | 212bp | [22] |
| (MAL13P1_475) | Pfhrp3_flak475_R | TCG TAC AAT TCA TCA TAC TCA CC | 55ºC x 50 sec, 72ºC x 1min; 72ºC x 5min |  |  |
| PF3D7_1372400 | Pfhrp3_flak485_F | TTGAGTGCAATGATGAGTGGAG | 94ºC x 5 min, 30 cycles of 94ºC x 30 sec, | 241bp | [22] |
| (MAL13P1_485) | Pfhrp3_flak485_R | AAATCATTTCCTTTTACACTAGTGC | 60ºC x 50 sec, 72ºC x 1min; 72ºC x 5min |  |  |
