# Supplementary material for: Detection of high prevalence of Plasmodium falciparum histidine-rich protein 2/3 gene deletions in Assosa zone, Ethiopia: implication for malaria diagnosis
Source: Malar J. 2021 Feb 23;20:109. doi: 10.1186/s12936-021-03629-x (PMC8095343; doi:10.1186/s12936-021-03629-x)
Supplement: Supplementary file 3 — Additional file 3: Deletion pattern of Pfhrp2/3 and their respective flanking regions among 218 samples. [file 12936_2021_3629_MOESM3_ESM.docx]

**Additional file 3.Deletion pattern of *Pfhrp2/3* and their respective flanking regions among 218 samples**

|  | | | | |  |  |  |  |
| --- | --- | --- | --- | --- | --- | --- | --- | --- |
| *MAL7P1_*  *230* | *pfhrp2 exon E1-2* | *Pfhrp2 Exon2* | *MAL7P1_*  *228* | *MAL13P1_*  *475* | *Pfhrp3 Exon1-2* | *Pfhrp3 exon2* | *MAL13P1_*  *485* | Sample No |
| + | + | + | + | + | + | + | + | 81 |
| + | + | + | + | + | + | + | - | 2 |
| + | + | + | + | + | + | - | + | 8 |
| + | + | + | + | - | + | + | - | 1 |
| + | + | + | - | + | + | + | + | 3 |
| + | + | + | - | - | + | + | - | 3 |
| + | + | + | - | + | + | + | - | 1 |
| + | + | + | - | - | + | - | - | 1 |
| + | - | + | + | + | + | + | + | 2 |
| + | - | + | + | + | + | - | + | 1 |
| + | + |  | - | - | + | + | - | 1 |
| + | - | + | - | - | + | + | - | 13 |
| + | - | - | - | - | + | + | - | 5 |
| + | + | + | + | - | - | - | - | 1 |
| + | - | + | + | - | + | + | - | 1 |
| + | - | + | - | - | + | + | + | 1 |
| - | + | + | + | + | + | + | + | 1 |
| - | - | + | - | - | + | + | - | 46 |
| - | + | + | - | + | + | + | + | 3 |
| - | + | + | - | - | + | + | - | 3 |
| - | - | + | - | + | - | - | + | 1 |
| - | - | + | - | + | + | - | - | 1 |
| - | - | - | - | + | + | + | - | 1 |
| - | - | - | - | - | + | + | - | 26 |
| - | - | + | - | - | - | + | - | 3 |
| - | - | - | - | - | - | + | - | 1 |
| - | - | - | - | - | + | - | - | 3 |
| - | - | + | - | - | - | - | - | 1 |
| - | - | + | - | - | + | - | - | 1 |
| - | - | - | - | - | - | - | - | 2 |
| Total Number | | | | | | | | 218 |

**Note:**+= PCR positive/gene present, - =PCR negative/deletion of gene**.** Upstream *(PF3D7_0831900(MAL7P1_230)*anddownstream *(PF3D7_0831700(MAL7P1_228)* flanking regions of *Pfhrp2*. Upstream*(PF3D7_1372100(MAL13P1_475))* and*)*downstream*(PF3D7_1372400(MAL13P1_485))*flanking regions of *pfhrp3*
