# Supplementary material for: Detection of high prevalence of Plasmodium falciparum histidine-rich protein 2/3 gene deletions in Assosa zone, Ethiopia: implication for malaria diagnosis
Source: Malar J. 2021 Feb 23;20:109. doi: 10.1186/s12936-021-03629-x (PMC8095343; doi:10.1186/s12936-021-03629-x)
Supplement: Supplementary file 4 — Additional file 4: The results of PCR Pfhrp2 exon2, PfHRP2 RDTs, Microscopy with Parasite density /µl, and qPCR with parasite density/μl and Ct values among P.falciparum positive isolates. [file 12936_2021_3629_MOESM4_ESM.docx]

**Additional file 4.** The results of PCR *Pfhrp2* *exon2*, PfHRP2 RDTs, Microscopy with Parasite density /µl, and qPCR with parasite density/μl and Ct values among *P.falciparum* positive isolates

|  | **Laboratory results** | | |  | |
| --- | --- | --- | --- | --- | --- |
| **Sample I.D.** | ***Pfhrp2 exon2(PCR)*** | **PfHRP2-RDT** | **Microscopy/ Parasite density /µl** | **Ct-value** | **Parasite density/µl (qPCR)** |
| HShr20 | - | - | - | 35.88 | 27.05 |
| HShr56 | - | - | - | 35.77 | 27.81 |
| HShr60 | - | - | - | 34.68 | 78.32 |
| HShr74 | - | - | - | 36.11 | 20.13 |
| HShr76 | - | - | - | 35.79 | 27.29 |
| HShr112 | - | - | - | 34.13 | 132.07 |
| HShr125 | - | - | - | 35.43 | 38.41 |
| HShr130 | - | - | - | 36.1 | 20.33 |
| HShr150 | - | - | - | 35.68 | 30.29 |
| HShr154 | - | - | +/4800 | 31.64 | 1406.49 |
| HBab27 | - | - | - | 36.12 | 19.94 |
| HBab63 | - | - | - | 36.63 | 12.28 |
| HBab83 | - | - | - | 33.11 | 348.05 |
| HBab90 | - | - | - | 35.35 | 41.44 |
| HBab91 | - | - | - | 33.82 | 177.3 |
| HKum10 | - | - | - | 31.6 | 1460.97 |
| HKum14 | - | - | - | 36.91 | 9.42 |
| HKum45 | - | - | - | 21.92 | 14401642.48 |
| HKum55 | - | - | - | 24.98 | 786897.14 |
| HKum68 | - | - | - | 21.39 | 23827544.93 |
| HKum69 | - | - | - | 20 | 89241150.48 |
| HAss07 | - | - | - | 26.42 | 200356.35 |
| HAss09 | - | - | - | 33.53 | 233.54 |
| HAss19 | - | - | - | 35.32 | 42.64 |
| HAss25 | - | - | - | 35.89 | 24.81 |
| HAss30 | - | - | - | 35.81 | 26.77 |
| HAss45 | - | - | - | 35.73 | 28.89 |
| LShr69 | - | - | - | 35.58 | 33.31 |
| LShr106 | - | - | - | 34.11 | 134.61 |
| LShr180 | - | - | - | 33.93 | 159.71 |
| LBab16 | - | - | - | 32 | 999.1 |
| LBab30 | - | - | +/1200 | 35.59 | 32.99 |
| LBab 65 | - | - | - | 35.59 | 32.99 |
| LBab78 | - | - | - | 35.73 | 28.89 |
| LKum16 | - | - | - | 35 | 57.79 |
| LKum59 | - | - | +/10000 | 32.27 | 773.06 |
