# Supplementary material for: Detection of high prevalence of Plasmodium falciparum histidine-rich protein 2/3 gene deletions in Assosa zone, Ethiopia: implication for malaria diagnosis
Source: Malar J. 2021 Feb 23;20:109. doi: 10.1186/s12936-021-03629-x (PMC8095343; doi:10.1186/s12936-021-03629-x)
Supplement: Supplementary file 5 — Additional file 5: Pfhrp2 exon2 negatives, pfhrp3 exon2 , PfHRP2 RDT and microscopy positive samples. [file 12936_2021_3629_MOESM5_ESM.docx]

| **Additional file 5:*Pfhrp2* exon2 negatives, *pfhrp3* exon2 , PfHRP2 RDT and microscopy positive samples** | | | | | | | | | |  |  |
| --- | --- | --- | --- | --- | --- | --- | --- | --- | --- | --- | --- |
| ID | Pfhrp2 exon2 | Pfhrp2 Exon1-2 | Pfhrp3 exon2 | Pfhrp3 Exon1-2 | PfHRP2-RDT | *MAL7P1*  *_228* | *MAL7P1*  *_230* | *MAL13P1*  *_475* | *MAL13P1*  *_485* | Microscopy | Parasitemia |
| HShr142 | - | - | + | + | + | - | - | - | - | + | 11960 |
| HKum22 | - | + | + | + | + | - | + | - | - | + | 11960 |
| HKum33 | - | - | + | + | + | - | + | - | - | + | 11520 |

Note: Upstream *(PF3D7_0831900(MAL7P1_230)*and downstream *(PF3D7_0831700(MAL7P1_228)* flanking regions of *Pfhrp2*. Upstream*(PF3D7_1372100(MAL13P1_475))* and*)*downstream*(PF3D7_1372400(MAL13P1_485))*flanking regions of *pfhrp3*
