# Supplementary material for: Detection of high prevalence of Plasmodium falciparum histidine-rich protein 2/3 gene deletions in Assosa zone, Ethiopia: implication for malaria diagnosis
Source: Malar J. 2021 Feb 23;20:109. doi: 10.1186/s12936-021-03629-x (PMC8095343; doi:10.1186/s12936-021-03629-x)
Supplement: Supplementary file 6 — Additional file 6: PfHRP2 RDT and microscopy-based results against PCR-based results of pfhrp2 and pfhrp3 their flanking regions. [file 12936_2021_3629_MOESM6_ESM.docx]

| **Additional file 6. PfHRP2 RDT and microscopy-based results against PCR-based results of pfhrp2 and pfhrp3 their flanking regions** | | | | | | | | | |
| --- | --- | --- | --- | --- | --- | --- | --- | --- | --- |
|  | | **upstream flanking region** | | | | **downstream flanking region** | | | |
|  |  | *MAL7P1_230* | | *MAL13P1_475* | | *MAL7P1_228* | | *MAL13P1_485* | |
|  |  | Pos. No. | Neg. No. | Pos. No. | Neg. No. | Pos. No. | Neg. No. | Pos No. | Neg. No. |
| PfHRP2 RDT-based results | Positive | 108 | 24 | 97 | 35 | 95 | 37 | 96 | 36 |
|  | Negative | 17 | 69 | 8 | 78 | 3 | 83 | 5 | 81 |
| Microscopy-based results | Positive | 107 | 24 | 98 | 33 | 95 | 36 | 96 | 35 |
|  | Negative | 18 | 69 | 7 | 80 | 3 | 84 | 5 | 82 |

Neg. (negative); Pos. (positive)
